# Prognostic Nutritional Index, Another Prognostic Factor for Extranodal Natural Killer/T Cell Lymphoma, Nasal Type

**DOI:** 10.3389/fonc.2020.00877

**Published:** 2020-06-19

**Authors:** Ningning Yao, Qing Hou, Shuangping Zhang, Huan Xiao, Yu Liang, Xiaokai Xu, Ruyuan Guo, Hongwei Li, Shengmin Lan, Hongwei Si, Jianzhong Cao

**Affiliations:** ^1^Department of Radiobiology, Shanxi Provincial Cancer Hospital, Taiyuan, China; ^2^Department of Surgery, Shanxi Provincial Cancer Hospital, Taiyuan, China; ^3^Department of Nuclear Medicine, The First Affiliated Hospital of Anhui Medical University, Hefei, China; ^4^Department of Radiotherapy, Shanxi Provincial Cancer Hospital, Taiyuan, China

**Keywords:** extranodal natural killer/T cell lymphoma, nasal type, prognostic nutritional index, lymphocyte, albumin, prognosis

## Abstract

**Objective:** The prognostic nutritional index (PNI) is a significant prognostic factor in diffuse large B cell lymphoma, follicular lymphoma, and other malignancies. The current study aimed to explore its prognostic role in extranodal natural killer/T cell lymphoma (ENKTL).

**Methods:** Patients diagnosed with ENKTL and treated during 2002 and 2018 (*n* = 184) were retrospectively recruited. PNI was calculated from albumin concentration (g/L) and total lymphocyte count (^*^10^9^/L). The association of PNI and overall survival (OS) or progression-free survival (PFS) was assessed in univariate analysis and multivariate Cox regression validated by the 10-fold cross-validation method.

**Results:** Survival analyses showed that both OS and PFS differed significantly between PNI groups stratified by a cutoff value of 49.0. The 3- and 5-year OS were 42.5 and 36.3% in the low-PNI (PNI < 49) subgroup and 70.6% and 63.9% (*P* < 0.001) in the high-PNI (PNI ≥ 49) subgroup, respectively. The corresponding PFS showed a similar pattern (38.4, 32.4 vs. 64.8, 54.0%, *P* < 0.001). Multivariate analysis indicated that PNI was significantly independent for both OS (HR = 0.517, 95% CI = 0.322–0.831, *P* = 0.006) and PFS (HR = 0.579, 95% CI = 0.373–0.899, *P* = 0.015). Furthermore, integrating PNI into the models of IPI (International Prognostic Index), KPI (Korean Prognostic Index), and PINK (prognostic index of natural killer lymphoma) could improve the area under the curve (AUC) and reduce the integrated Brier score (IBS) and Akaike Information Criterion (AIC) value of each model.

**Conclusion:** PNI was a significant prognostic indicator for ENKTL.

## Introduction

As a rare non-Hodgkin's lymphoma, extranodal natural killer/T cell lymphoma, nasal type (ENKTL) is closely associated with Epstein–Barr virus infection and relatively prevalent in Asia and South America (1, 2). Although the patients' prognosis has been obviously improved by new drugs and radiotherapy techniques (3, 4), some of them are still frequently relapsed.

Previous studies indicated the compromised prognostic role of international prognostic index (IPI) in ENKTL compared to other aggressive non-Hodgkin's lymphomas (5, 6). Therefore, the Korean Prognostic Index (KPI) was proposed, and its prognostic power could be further improved by integrating some laboratory results (7–10). After that, other predictors were explored, such as regional lymph node involvement, primary tumor invasion, hemoglobin level, and others (11–13).

It was reported that the progression of cancer was strongly associated with inflammation and nutritional status (14). Although the molecular mechanism was still unclear, many nutrition and inflammatory factors were related to the prognosis of cancer patients (9, 15, 16). The prognostic nutritional index (PNI), calculated from serum albumin (17) and absolute peripheral lymphocyte count (18), is an integrated factor for both nutritional status and systemic inflammation. It was a prognostic marker for some malignancies, including diffuse large B cell lymphoma and follicular lymphoma (19–21). Therefore, in this study, we tried to evaluate its prognostic ability among ENKTL patients.

## Materials and Methods

### Patients and Method

From Jan 2002 to Dec 2018, ENKTL (nasal type) patients were retrospectively retrieved from the documents at the Shanxi Provincial Cancer Hospital. The inclusion criteria were as follows: (a) pathologically proved ENKTL according to the WHO Classification of Tumors of Haematopoietic and Lymphoid Tissues, (b) no anti-tumor treatment before diagnosis, (c) with follow-up >1 month. Patients with upper and non-upper aerodigestive tract NK/T-cell lymphoma (UADT and non-UADT) were stratified according to the previous studies (22). Primary tumor invasion (PTI) was assessed according to the published criteria (23). The followed prognostic factors included those in the models of IPI (24), KPI (5), PIT (25), and PINK (26), and sex, laboratory measurements, etc. The study was approved by the ethics committees at the Shanxi Provincial Cancer Hospital, and the review board approved to waive the requirement for informed consent.

### Laboratory Measurements

Blood cells and serum albumin level were quantified by the Sysmex XE-5000 fully automated hematological analyzer (Japan) and the Hitachi 7600 automatic biochemical analyzer (Japan), respectively. The reports included white blood cell (WBC), neutrophil, monocyte, lymphocyte, and platelet count, hemoglobin, and serum albumin levels. PNI was calculated by Equation (1) (27).

(1) $$\begin{array}{r} \text{PNI }=\text{ albumin }(\text{g/L})\;+5\times \text{ lymphocyte count }(*10^{9}\text{/L}) \end{array}$$

### Statistical Analysis

The optimal cutoff value for stratifying PNI was determined by the change point method from Contal and O'Quigley (SurvMisc package, R project, version 3.6.1) (28). The potentially confounded factors between the high- and low-PNI groups were balanced by the propensity score matching (PSM) method with a ratio of 1:1 and a caliper width of 0.5 (Matchit package, R project, version 3.6.1) (29). The matched factors included treatment modalities, age, sex, ECOG score, Ann Arbor Stage, B symptoms, LDH level, regional lymph node involvement, subtype, and extranodal sites of involvement. Between the matched PNI stratifications, patient characteristics were in Supplementary Table 1, and the univariate and multivariate analysis against OS/PFS were performed.

Overall survival (OS) was defined from the date of diagnosis to death from any reasons. Progression-free survival (PFS) was measured from the date of diagnosis to the first relapse, progression, or death. The survival differences between the stratifications of prognostic factors were analyzed by the Kaplan–Meier method and log-rank test, and the effect of risk factors on survival was analyzed by the univariate and multivariate Cox proportional hazards regression (*P* < 0.05). The regression was confirmed by the 10-fold cross-validation method (30) and was evaluated by the indices of AUC and integrated Brier score (IBS), respectively. IBS is an index of prediction error, and a lower value indicates better accuracy. Akaike Information Criterion (AIC) analysis was also performed to compare the discriminative abilities of different models, and a predictive model with a low smaller AIC value indicates a better model fit.

## Results

### Patient Characteristics

Patient characteristics are listed in Table 1 (*n* = 184). The median age of the patients is 46 years (range: 9–81 years). In this cohort of patients, the optimal cutoff value of PNI was determined by the change point method and was comparable to that in the previous study (49 vs. 45) (31). The low- and high-PNI stratifications had diverse baseline characteristic distributions in ECOG, LDH level, hemoglobin, neutrophil, lymphocytes, and white blood cell count, and received significant treatment strategies. Characteristics of the patients matched by the PSM method are listed in Supplementary Table 1. Except KPI (*P* = 0.046), almost all the variables are well-balanced between the PNI stratifications.

**Table 1:** Comparison of patient characteristics between the PNI stratifications.

**Characteristics**	**All**	**PNI < 49**	**PNI ≥ 49**	***P***
	**No. (%)**	**No. (%)**	**No. (%)**	
**Age**				
≤60 years	148 (80.4)	72 (75.8)	76 (85.4)	0.101
>60 years	36 (19.6)	23 (24.2)	13 (14.6)	
**Sex**				
Male	145 (78.8)	70 (73.7)	75 (84.3)	0.079
Female	39 (21.2)	25 (26.3)	14 (15.7)	
**ECOG score**				
0–1	147 (79.9)	70 (73.7)	77 (86.5)	0.030
≥2	37 (20.1)	25 (26.3)	12 (13.5)	
**LDH level**				
≤245 U/L	130 (70.7)	60 (63.2)	70 (78.7)	0.021
>245 U/L	54 (29.3)	35 (36.8)	19 (21.3)	
**B symptoms**				
No	117 (63.6)	55 (57.9)	62 (69.7)	0.097
Yes	67 (36.4)	40 (42.1)	27 (30.3)	
**No. of extranodal sites**				
<2	159 (86.4)	78 (82.1)	81 (91.0)	0.078
≥2	25 (13.6)	17 (17.9)	8 (9.0)	
**Ann Arbor Stage**				
I–II	142 (77.2)	68 (71.6)	74 (83.1)	0.062
III–IV	42 (22.8)	27 (28.4)	15 (16.9)	
**Primary site**				
UADT	170 (92.4)	88 (92.6)	82 (92.1)	0.899
Non-UADT	14 (7.6)	7 (7.4)	7 (7.9)	
**PTI**				
Absent	118 (64.1)	56 (58.9)	62 (69.7)	0.130
Present	66 (35.9)	39 (41.1)	27 (30.3)	
**KPI score**				
0–1	117 (63.6)	50 (52.6)	67 (75.3)	0.001
2–4	67 (36.4)	45 (47.4)	22 (24.7)	
**PIT score**				
0–1	154 (83.7)	72 (75.8)	82 (92.1)	0.003
2–4	30 (16.3)	23 (24.2)	7 (7.9)	
**IPI score**				
0–1	133 (72.3)	61 (64.2)	72 (80.9)	0.011
2–5	51 (27.7)	34 (35.8)	17 (19.1)	
**PINK score**				
0	100 (54.3)	45 (47.4)	55 (61.8)	0.139
≥1	84 (45.7)	50 (52.7)	34 (38.2)	
**Leukocyte**				
≤1.5 × 10^9^/L	86 (46.7)	71 (74.7)	15 (16.9)	<0.001
>1.5 × 10^9^/L	98 (53.3)	24 (25.3)	74 (83.1)	
**Neutrophil**				
≤3.0 × 10^9^/L	83 (45.1)	56 (58.9)	27 (30.3)	<0.001
>3.0 × 10^9^/L	101 (54.9)	39 (41.4)	62 (69.7)	
**Platelet**				
≤200 × 10^9^/L	71 (38.6)	40 (42.1)	31 (34.8)	0.311
>200 × 10^9^/L	113 (61.4)	55 (57.9)	58 (65.2)	
**White cell**				
≤5.0 × 10^9^/L	88 (47.8)	64 (67.4)	24 (27.0)	<0.001
>5.0 × 10^9^/L	96 (52.2)	31 (32.6)	65 (73.0)	
**Hemoglobin**				
≤120 g/L	44 (23.9)	33 (34.7)	11 (12.4)	<0.001
>120 g/L	140 (76.1)	62 (65.3)	78 (87.6)	
**Treatment**				
RT alone	15 (8.2)	8 (8.4)	7 (7.9)	0.097
CT alone	61 (33.1)	38 (40.0)	23 (25.8)	
CRT	108 (58.7)	49 (51.6)	59 (66.3)	
**CT regimen**				
L-Asp-based	83 (49.1)	45 (51.7)	38 (46.3)	0.539
Other	86 (50.9)	42 (48.3)	44 (53.7)	

*UADT, upper aerodigestive tract NK/T-cell lymphoma; PTI, primary tumor invasion; KPI, Korean Prognostic Index; IPI, International Prognostic Index; PINK, Prognostic index of natural killer lymphoma; LDH, lactate dehydrogenase; RT, radiotherapy; CT, chemotherapy; CRT, chemoradiotherapy; L-Asp, L-asparaginase*.

### Treatment Strategy

The enrolled patients were treated by radiotherapy alone (*n* = 15), chemotherapy alone (*n* = 61), chemotherapy followed by radiotherapy (*n* = 83), radiotherapy followed by chemotherapy (*n* = 11), and concurrent radio-chemotherapy (*n* = 14). Initial chemotherapy regimens were CHOP or CHOP-like (*n* = 39), L-Asparaginase (L-Asp) (*n* = 83), gemcitabine (*n* = 28), and others (*n* = 19). The treatment modalities and chemotherapy regimens were not significantly different between the PNI stratifications (Table 1).

### Survival

**Figure 1:**
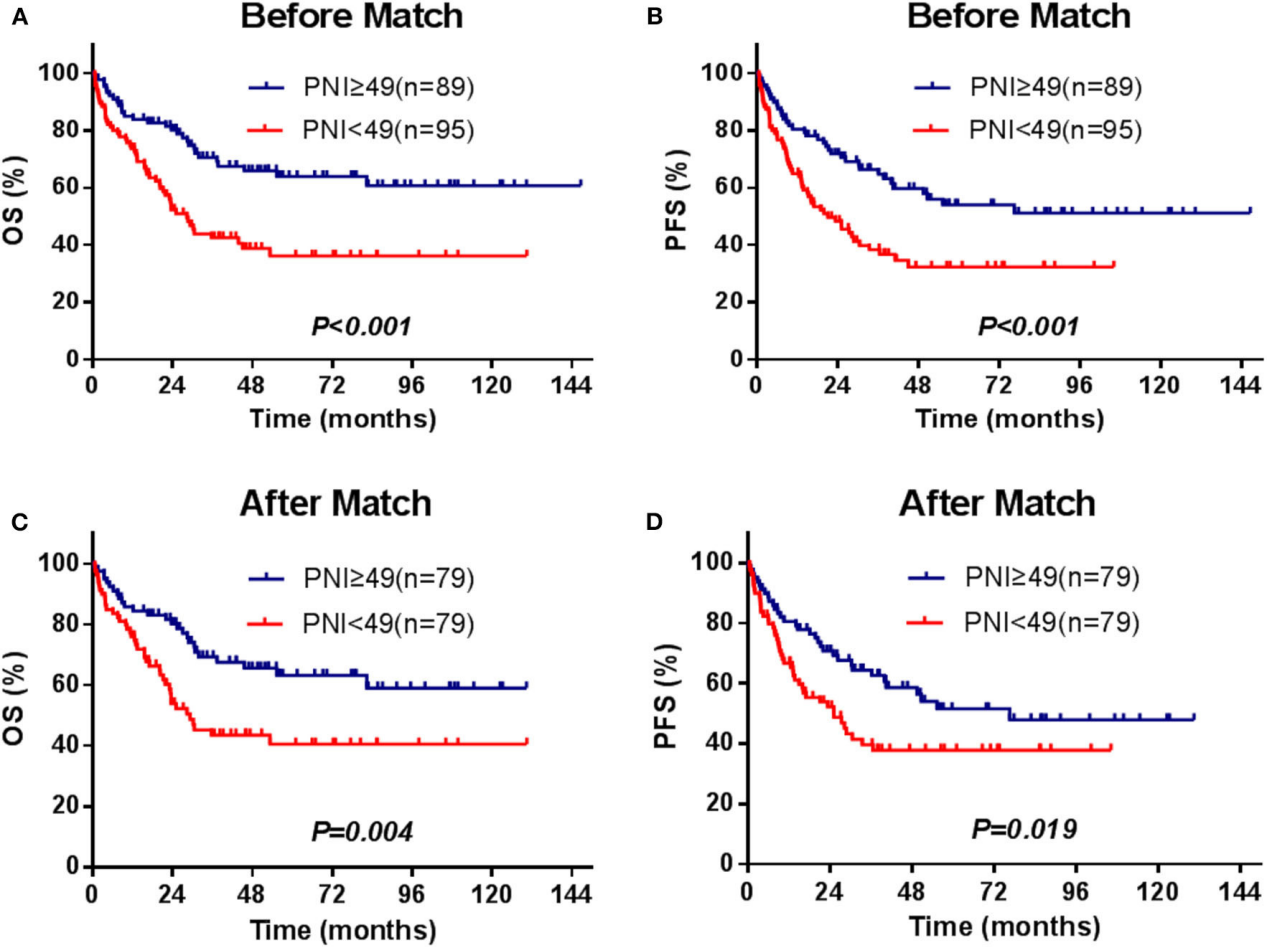
Survival curves for the PNI stratifications (≥49, <49), OS, and PFS for the enrolled patients **(A,B)** and for those[[Inline Image]] matched by the PSM method **(C,D)**.

The median survival time of the patients was 82.4 months, and 82 patients (44.6%) died during the period of follow-up. The 3- and 5-year OS were 56.3 and 50.1%, and 3- and 5-year PFS were 52.1 and 42.9%, respectively. The significant factors of OS or PFS with univariate survival analysis are listed in Table 2. The high-PNI group had higher 5-year OS (63.9 vs. 36.3%; *P* < 0.001) and PFS (54.0 vs. 32.4%; *P* < 0.001) than the low-PNI group (Figures 1A,B).

**Table 2:** Univariate survival analysis of the enrolled patients.

**Factors**		**OS (%)**		***P***	**PFS (%)**		***P***
		**3 years**	**5 years**		**3 years**	**5 years**	
Age	≤60 years	58.8	53.6	0.032	53.6	45.7	0.132
	>60 years	45.8	35.5		46.0	31.5	
Sex	Male	58.0	51.7	0.441	52.8	45.2	0.382
	Female	49.6	44.7		49.9	30.4	
ECOG score	0–1	64.6	57.8	<0.001	60.9	49.5	<0.001
	≥2	25.9	22.2		18.5	18.5	
LDH level	≤245 U/L	60.4	57.9	0.002	56.0	48.6	0.013
	>245 U/L	46.8	25.7		36.8	26.8	
B symptoms	No	54.6	48.6	0.669	51.9	41.5	0.980
	Yes	59.7	52.9		52.7	45.5	
No. of extranodal sites	<2	58.8	52.0	0.077	54.2	44.0	0.158
	≥2	38.9	38.9		39.1	39.1	
Ann Arbor Stage	I–II	59.4	52.0	0.245	53.9	43.2	0.561
	III–IV	44.6	44.6		45.3	45.3	
Primary site	UADT	56.2	49.6	0.541	51.6	43.8	0.787
	Non-UADT	58.0	58.0		58.0	21.8	
Regional lymph node involvement	No	63.0	55.3	0.004	57.6	47.5	0.008
	Yes	31.3	31.3		27.6	27.6	
PNI	PNI < 49	42.5	36.3	<0.001	38.4	32.4	0.001
	PNI≥49	70.6	63.9		66.3	54.0	
KPI score	0–1	61.9	56.8	0.012	57.6	47.7	0.029
	2–4	45.7	35.2		41.5	33.7	
PIT score	0–1	62.1	56.8	<0.001	57.7	48.1	<0.001
	2–5	27.4	27.4		24.1	24.1	
IPI score	0–1	62.5	56.8	0.001	57.5	47.3	0.011
	2–5	39.3	28.0		37.1	29.7	
PINK score	0	63.5	56.5	0.014	55.9	47.5	0.097
	≥1	46.7	41.2		44.8	36.1	
Leukocytopenia	≤1.5 × 10^9^/L	45.4	36.6	0.002	41.0	32.2	0.002
	>1.5 × 10^9^/L	65.7	61.0		61.7	51.9	
Neutropenia	≤3.0 × 10^9^/L	50.4	40.1	0.092	45.0	33.8	0.115
	>3.0 × 10^9^/L	61.3	58.1		57.9	49.9	
Platelets	≤200 × 10^9^/L	47.7	45.6	0.240	44.9	42.8	0.416
	>200 × 10^9^/L	61.9	52.7		56.6	42.1	
White cell count	≤5.0 × 10^9^/L	50.5	46.5	0.189	45.7	40.8	0.194
	>5.0 × 10^9^/L	61.5	53.6		56.5	45.2	
Hemoglobin	≤120 g/L	47.4	41.0	0.136	45.5	27.1	0.138
	>120 g/L	59.3	53.4		54.3	47.3	
RT	Yes	63.1	56.1	0.005	58.5	50.3	0.002
	No	42.8	38.1		39.1	28.5	
L-Asp-based CT	Yes	65.1	61.0	0.004	61.2	51.1	0.009
	No	49.1	42.0		44.7	36.0	

*UADT, upper aerodigestive tract NK/T-cell lymphoma; KPI, Korean Prognostic Index; IPI, International Prognostic Index; PINK, Prognostic index of natural killer lymphoma; LDH, lactate dehydrogenase; RT, radiotherapy; L-Asp, L-asparaginase; CT, chemotherapy*.

**Figure 2:**
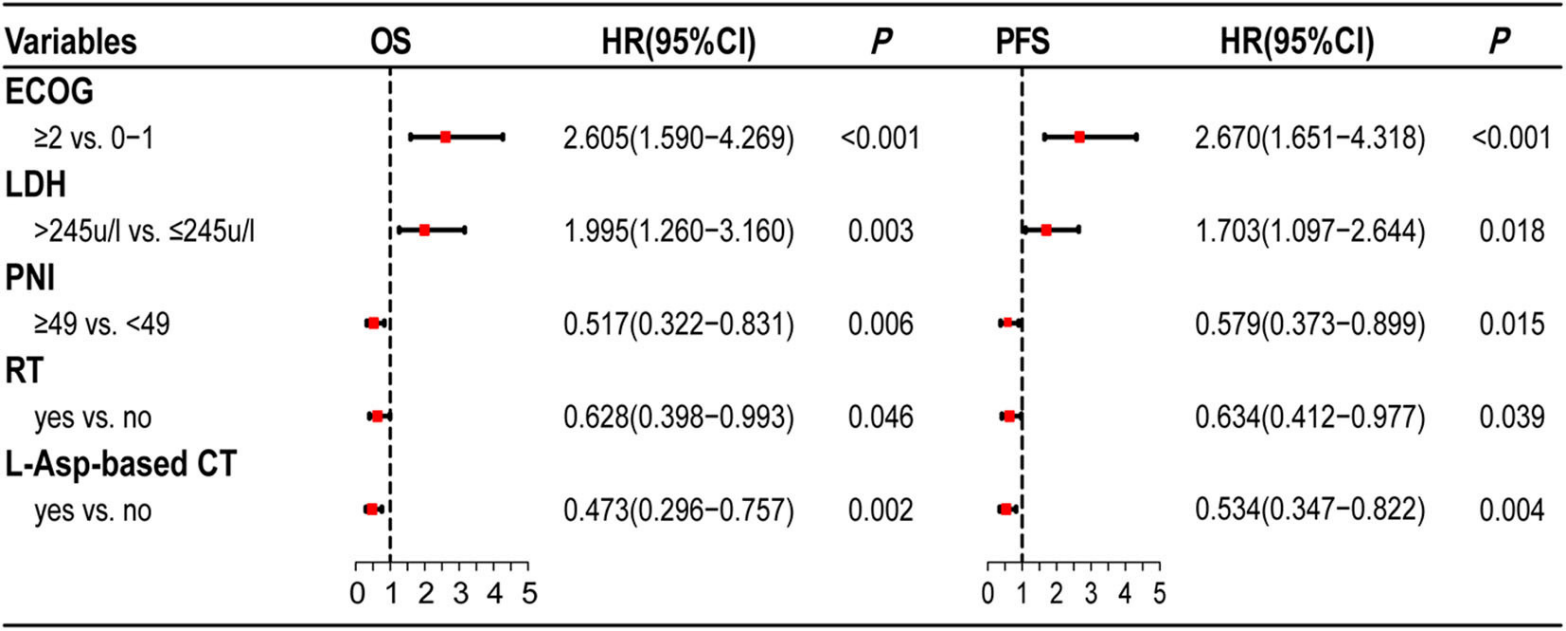
Forest plots of multivariate analysis. PNI is as an independently prognostic factor for OS and PFS.

The multivariate survival analysis validated by the 10-fold cross method indicated that PNI was an independently prognostic predictor for both OS (HR = 0.517, 95% CI = 0.322–0.831, *P* = 0.006) and PFS (HR = 0.579, 95% CI = 0.373–0.899, *P* = 0.015). The prognostic effect of significant factors with multivariate analysis (PNI, ECOG score, serum LDH level, radiotherapy, and L-Asp-based chemotherapy) was demonstrated by the forest plot (Figure 2).

In the univariate and multivariate analysis of the PSM patients (Supplementary Table 2), PNI was also significantly against OS (HR = 0.522, 95% CI = 0.318–0.858, *P* = 0.010) and PFS (HR = 0.609, 95% CI =0.385–0.963, *P* = 0.034). The adjusted 5-year OS and PFS were 77.8 and 62.9% in the high-PNI group and were 54.3 and 40.7% in the low-PNI group (Figures 1C,D), respectively. In the Cox regression validated by the 10-fold cross method, the factors PNI, ECOG, PIT score, and L-Asp-based chemotherapy were significant for both OS and PFS. Above all, whether PSM is balanced or not, PNI was a significant factor for the prognosis of the patients.

### PNI Under L-Asp-Based Chemotherapy

**Figure 3:**
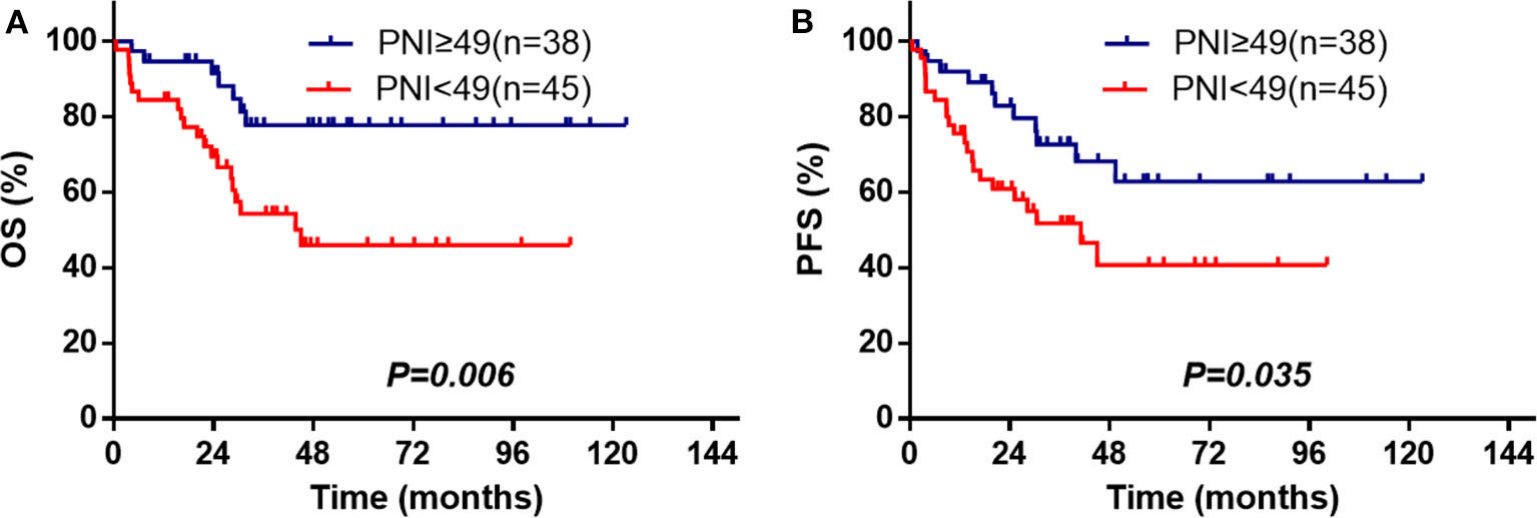
PNI is associated with OS **(A)** and PFS **(B)** among the ENKTL patients treated with L-Asp-based chemotherapy.

In this study, 83 patients were treated with L-Asp-based chemotherapy with/without RT. Among these patients, the 3- and 5-year OS (*n* = 45) were 54.3 and 45.9% in the low-PNI group and were 77.8 and 77.8% in the high-PNI group (*n* = 38; Figure 3A), respectively. Similar results were also found in PFS (Figure 3B). The multivariate analysis indicated that PNI was also significantly against OS (HR = 0.327, 95% CI = 0.137–0.782, *P* = 0.012) and PFS (HR = 0.461, 95% CI = 0.221–0.961, *P* = 0.039; Supplementary Table 3).

### Prognostic Power of PNI

**Figure 4:**
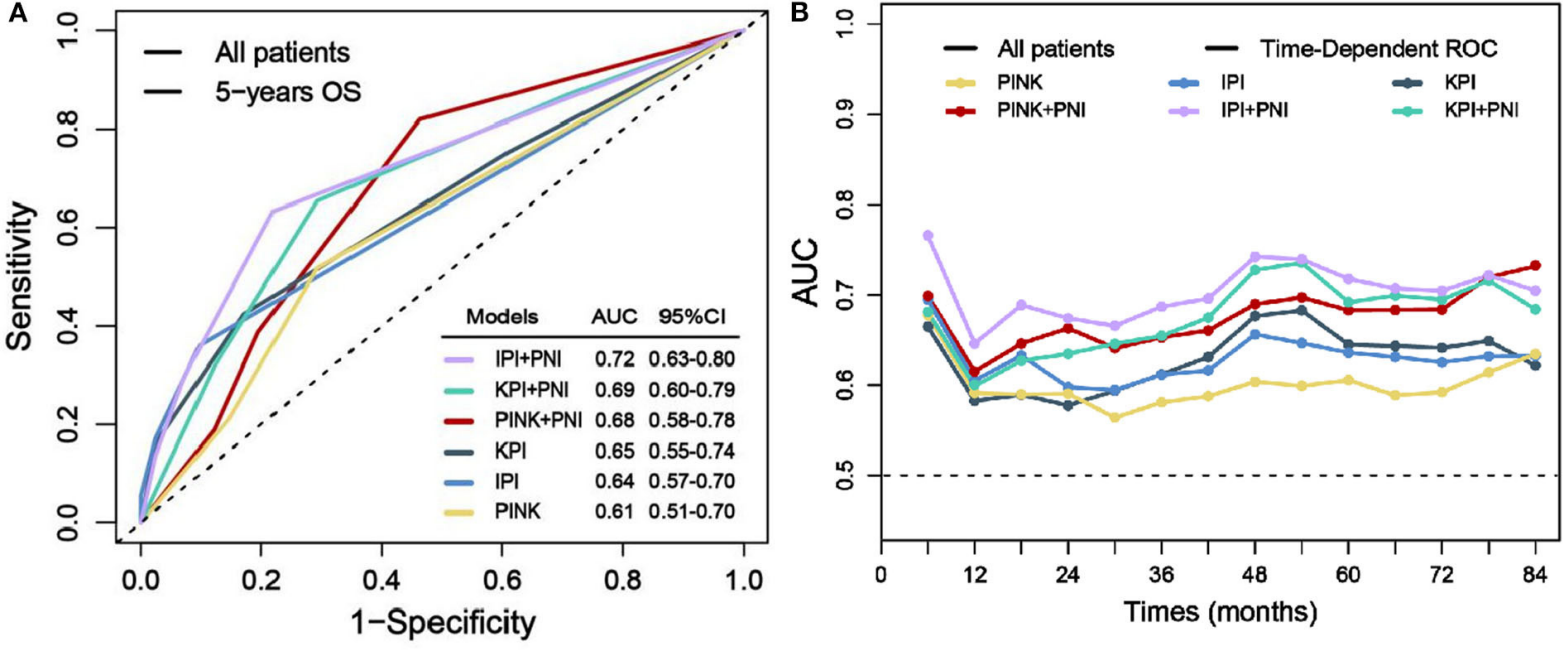
Prognostic power evaluation of PNI. Before and after integrating PNI, AUC of the models in predicting 5-year OS **(A)** and the time-dependent AUC **(B)**.

To evaluate the prognostic power of PNI in ENKTL patients, we separately integrate PNI into the model of IPI, KPI, and PINK, which are defined in Supplementary Table 4. Figure 4A demonstrates that after integrating PNI, the area under the curve (AUC) of all models could be improved from 4 to 8%, and the results are confirmed by the time-dependent AUC (Figure 4B). Furthermore, IBS values of IPI, KPI, and PINK could be decreased from 0.190 to 0.183, from 0.196 to 0.191, and from 0.203 to 0.194 (Supplementary Figure 1). The AIC value of the models were also decreased from 776.46 to 770.29, from 783.73 to 779.72, and from 787.41 to 780.56, respectively. Therefore, integrating PNI into current prognostic models could improve AUC, reduce estimation error (as indicated by IBS), and showed better predictive abilities (as indicated by a low AIC value) in this cohort.

## Discussion

In this study, PNI related to the prognosis of ENKTL patients could be used to improve the performance of the frequently used models. Recently, PNI was identified as a prognostic marker for some malignancies; however, only the study from Gallamini et al. (25) was on ENKTL. Therefore, additional studies should be performed to verify the correlation between PNI and outcome of the patients. In this study, besides PNI, other valuable factors were also identified, such as ECOG score, LDH level, radiotherapy, and L-Asp-based chemotherapy for the ENKTL patients. Among them, it was necessary to further explore the efficacy of L-Asp-based chemotherapy.

In this study, PNI related to the prognosis of ENKTL patients could be used to improve the performance of the frequently used models. Recently, PNI was identified as a prognostic marker for some malignancies; however, only the study from Chen et al. (31) was on ENKTL. Therefore, additional studies should be performed to verify the correlation between PNI and outcome of the patients. In this study, besides PNI, other valuable factors were also identified, such as ECOG score, LDH level, radiotherapy, and L-Asp-based chemotherapy for the ENKTL patients. Among them, it was necessary to further explore the efficacy of L-Asp-based chemotherapy.

The cutoff value of PNI was usually estimated by the receiver operating characteristic curves (ROC) and was between 40 and 50 in previous studies (19, 21, 32, 33). However, if the censored observations that are ignored by the ROC method could be followed long enough, they would be eligible for the analysis (34). Therefore, to determine the optimal cutoff value of this cohort, we referred the method from Contal and O'Quigley (28) to include the potentially eligible cases.

Since ENKTL was a rare lymphatic disease, an external validation cohort was not easy to seek. We used the cross-validation method as a substitution, and our results indicated that PNI was a credible and powerful prognostic factor.

The efficacy of traditional chemotherapy, such as CHOP or CHOP-like regimens, was not so satisfactory for the treatment of ENKTL patients and local relapse frequently occurred (35, 36). Therefore, it is necessary to develop new and effective drugs. The L-Asp-based chemotherapy regimens were proposed for refractory and relapsed diseases, and a complete response (CR) rate of 55.6% was achieved (37). Additionally, a meta-analysis suggested that the L-Asp-based regimen could improve overall response rate (ORR) and CR for both localized and systemic ENKTL (38). In accordance with previous reports, our study showed that the L-Asp-based chemotherapy was significant against OS in multivariate Cox regression. Furthermore, among the patients who received L-Asp-based chemotherapy, PNI was still associated with both OS and PFS. Therefore, these results indicated the prognostic ability of PNI.

The Ann Arbor staging system, originally designed for Hodgkin's lymphoma, is conventionally used for predicting the prognosis of ENKTL and is debated for its applicable value. A study indicated no significant differences between early-stage and advanced diseases in the complete remission rate or in survival rates (39). Additionally, China and Asia Lymphoma Study Group reported that the survival of stage IV patients was even better than those in stage III (40). These results coincided with ours and might be explained by the highly unbalanced distribution that stage I/II patients occupied almost 80% of newly diagnosed cases (41, 42).

Currently, many prognostic models, including the frequently used IPI, KPI, and PINK, have been proposed for ENKTL. However, these models have some more or less flaws and shortcomings. The patients were predominantly young in early-stage disease and good performance status and were in the low-risk groups (0–1) according to the IPI or KPI classification. Therefore, in 2016, the PINK score was proposed as a new prognostic model; however, it was not so powerful in some cohorts (43, 44). Similarly, our results indicated that PINK was even inferior to IPI or KPI, and integrating PNI into the models could improve their predictive ability. Above all, PNI was an effective prognostic factor for ENKTL patients and might have a promising application in the future.

## Conclusion

PNI is an effective prognostic factor for ENKTL patients with extranodal natural killer/T cell lymphoma, nasal type.

## Data Availability Statement

The datasets generated for this study are available on request to the corresponding author.

## Ethics Statement

The studies involving human participants were reviewed and approved by the Ethics Committee of Shanxi Provincial Cancer Hospital (the ethics number is 2019091). Written informed consent for participants was not required for this study in accordance with the national legislation and the institutional requirements.

## Author Contributions

JC and HS: research design and guidance, data acquisition, analysis and evaluation of statistical results, critical revision of the manuscript, and study supervision. NY, QH, SZ, and HX: data acquisition, study concept and design, analysis and interpretation of data, statistical analysis, and drafting of the manuscript. XX, YL, and RG: data acquisition, statistical analysis, organizing of data and tables, and reference surveys. HL and SL: critical revision of the manuscript and approved final submission.

## Conflict of Interest

The authors declare that the research was conducted in the absence of any commercial or financial relationships that could be construed as a potential conflict of interest.
