# Supplementary material for: Prognostic Nutritional Index, Another Prognostic Factor for Extranodal Natural Killer/T Cell Lymphoma, Nasal Type
Source: Front Oncol. 2020 Jun 19;10:877. doi: 10.3389/fonc.2020.00877 (PMC7317673; doi:10.3389/fonc.2020.00877)
Supplement: Supplementary file 1 [file Data_Sheet_1.PDF]

Supplementary Material

Supplementary Tables

Supplementary Table 1 Characteristics of the patients matched by PSM

Characteristics	Number of patients (%)	PNI<49	PNI≥49	*P*
Age				
≤60 y	130（82.3）	64（81.0）	66（83.5）	0.677
>60 y	28 （17.7）	15（19.0）	13（16.5）	
Sex				
Male	126（79.7）	61（77.2）	65（82.3）	0.428
Female	32 （20.3）	18（22.8）	14（17.7）	
ECOG score				
0-1	131（82.9）	64（81.0）	67（84.8）	0.526
≥2	27 （17.1）	15（19.0）	12（15.2）	
Serum LDH				
≤245 u/l	117（74.1）	57（72.2）	60（75.9）	0.586
>245 u/l	41 （25.9）	22（27.8）	19（24.1）	
B symptoms				
No	98 （62.0）	46（58.2）	52（65.8）	0.325
Yes	60 （38.0）	33（41.8）	27（34.2）	
Extranodal sites of involvement				
<2	137（86.7）	66（83.5）	71（89.9）	0.241
≥2	21 （13.3）	13（16.5）	8 （10.1）	
Ann Arbor Stage				
I-II	121（76.6）	57（72.2）	64（81.0）	0.189
III-IV	37 （23.4）	22（27.8）	15（19.0）	
Subtype				
UNKTL	146（92.4）	74（93.7）	72（91.1）	0.548
EUNKTL	12 （7.6）	5 （6.3）	7 （8.9）	
Regional lymph node involvement				
No	98 （62.0）	46（58.2）	52（65.8）	0.325
Yes	60 （38.0）	33（41.8）	27（34.2）	
KPI score				
0-1	102（64.6）	45（57.0）	57（72.2）	0.046
2-4	56 （35.4）	34（43.0）	22（27.8）	
IPI score				
0-1	122（77.2）	60（75.9）	62（78.5）	0.704
2-5	36 （22.8）	19（24.1）	17（21.5）	
PIT score				
0-1	143（90.5）	71（89.9）	72（91.1）	0.786
2-4	15 （9.5）	8 （10.1）	7 （8.9）	
PINK score				
0	86 （54.4）	41（51.9）	45（57.0）	0.413
1	35 （22.2）	16（20.3）	19（24.1）	

≥2	37 （23.4）	22（27.8）	15（19.0）	
Treatment modalities				
RT alone	13 （8.2）	7 （8.9）	6 （7.6）	0.533
CT alone	52 （32.9）	29（36.7）	23（29.1）	
CRT	93 （32.9）	43（54.4）	50（63.3）	
CT regimen				
L-Asp-based	75 （51.7）	37（51.4）	38（52.1）	1.000
Other	70 （48.3）	42（48.6）	35（47.9）	

PNI, Prognostic nutritional index; ***LDH***, lactate dehydrogenase; ***ECOG***, Eastern Cooperative Oncology Group; ***IPI***, International Prognostic Index; ***KPI***, Korean Prognostic Index; ***PINK***, prognostic index of natural killer lymphoma; ***PTI***, Prognostic Index for T-cell lymphoma; ***RT***, radiotherapy; ***CT,*** Chemotherapy; ***CRT,*** Chemoradiotherapy; ***L-Asp***, L-Asparaginase.

Supplementary Table 2 Univariate and multivariate analysis of the PSM patients

**Factors**	**OS**			**PFS**		
	**Univariate analysis**	**Multivariate analysis**		**Univariate analysis**	**Multivariate analysis**	
	***p* value**	**RR(95%CI)**	***p* value**	***p* value**	**RR(95%CI)**	***p* value**
**Age**						
>60 y vs. ≤60 y	0.129			0.308		
**Sex**						
Female vs. Male	0.225			0.240		
**ECOG score**						
≥2 vs. 0-1	<0.001*	2.005(1.149-3.499)	0.014	<0.001*	2.194(1.290-3.729)	0.004
**Ann Arbor Stage**						
III-IV vs. I-II	0.294			0.664		
**B symptoms**						
yes vs. no	0.494			0.596		
**Extranodal sites of involvement**						
≥2 vs. <2	0.213			0.370		
**Regional lymph node involvement**						
yes vs. no	0.049*			0.110		
**Subtype**						
EUNKTL vs. UNKTL	0.571			0.897		
**KPI**						
2-4 vs. 0-1	0.123			0.229		
**PIT**						
2-4 vs. 0-1	<0.001*	2.481(1.268-4.854)	0.008	0.003*		
**IPI**						
2-5 vs. 0-1	0.071*			0.264		
**PINK**						
0						
1						
≥2	0.027*			0.123		
**Neutrophil**						
>3.0 x10^9/l vs. ≤3.0 x10^9/l	0.230			0.333		
**Baseline white cell count**						

>5.0 x10^9/l vs. ≤5.0 x10^9/l	0.533			0.601		
**Platelets**						
>200 x10^9/l vs. ≤200 x10^9/l	0.462			0.690		
**Serum LDH**						
>245u/l vs. ≤245u/l	0.085*			0.284		
**Hb**						
>120g/l vs. ≤120g/l	0.096*			0.155		
**PNI**						
≥49 vs. <49	0.004*	0.522(0.318-0.858)	0.010	0.019*	0.609(0.385-0.963)	0.034
**RT**						
received vs. unreceived	0.037*			0.015*	0.618(0.390-0.981)	0.041
**L-Asp-based CT**						
received vs. unreceived	0.003*	0.510(0.304-0.856)	0.011	0.003*	0.524(0.326-0.843)	0.008

PNI, Prognostic nutritional index; ***LDH***, lactate dehydrogenase; ***ECOG***, Eastern Cooperative Oncology Group; ***IPI***, International Prognostic Index; ***KPI***, Korean Prognostic Index; ***PINK***, Prognostic index of natural killer lymphoma; ***PTI***, Prognostic Index for T-cell lymphoma; ***RT***, radiotherapy; ***L-Asp***, L-Asparaginase; ***CT,*** Chemotherapy.

Supplementary Table 3 Univariate and multivariate analysis of the patients treated with L-Asp-based chemotherapy.

**Factors**	**OS**			**PFS**		
	**Univariate analysis**	**Multivariate analysis**		**Univariate analysis**	**Multivariate analysis**	
	***p* value**	**RR(95%CI)**	***p* value**	***p* value**	**RR(95%CI)**	***p* value**
Age	0.995			0.925		
Sex	0.910			0.571		
ECOG score	0.037*	2.324(0.972-5.556)	0.058	0.069*	2.139(0.917-4.993)	0.079
Ann Arbor Stage	0.072*			0.263		
B symptoms	0.211			0.384		
Extranodal sites of involvement	0.191			0.421		
Regional lymph node involvement	0.074*	2.233(1.026-4.864)	0.043	0.092*	1.980(0.961-4.079)	0.064
Subtype	0.990			0.785		
KPI	0.066*			0.127		
PIT	0.041*			0.086*		
IPI	0.037*			0.132		
PINK	0.637			0.971		
Neutrophils	0.808			0.437		
Baseline white cell count	0.129			0.123		
Platelets	0.572			0.903		

Serum LDH	0.039*			0.198		
Hb	0.778			0.853		
PNI	0.006*	0.327(0.137-0.782)	0.012	0.035*	0.461(0.221-0.961)	0.039
RT	0.094*			0.116		

PNI, Prognostic nutritional index; ***LDH***, lactate dehydrogenase; ***ECOG***, Eastern Cooperative Oncology Group; ***IPI***, International Prognostic Index; ***KPI***, Korean Prognostic Index; ***PINK***, Prognostic index of natural killer lymphoma; ***PTI***, Prognostic Index for T-cell lymphoma; ***RT***, radiotherapy.

Supplementary Table 4 The variables and definitions of different models.

**Model and definition (total point)**	**Variable**	**point**
KPI		
Group1 (0)	Ann Arbor stage (III-IV vs. I-II)	1
Group2 (1)	Elevated LDH (yes vs. no)	1
Group3 (2)	B symptoms (yes vs. no)	1
Group4 (≥3)	Regional lymph node (yes vs. no)	1
IPI		
Low (0-1)	Age (>60 years vs. ≤60 years)	1
Intermediate low (2)	Ann Arbor stage (III-IV vs. I-II)	1
Intermediate high (3)	ECOG score (≥2 vs. 0-1)	1
High (≥4)	Elevated LDH (yes vs. no)	1
	Distant extranodal involvement (≥2 vs. 0-1)	1
PINK		
Low risk (0)	Age (>60 years vs. ≤60 years)	1
Intermediate risk (1)	Ann Arbor stage (III-IV vs. I-II)	1
High risk (≥2)	Distant extranodal involvement (yes vs. no)	1
	Nonnasal disease (yes vs. no)	1
KPI+PNI		
Group1 (0)	Ann Arbor stage (III-IV vs. I-II)	1
Group2 (1)	Elevated LDH (yes vs. no)	1
Group3 (2)	B symptoms (yes vs. no)	1
Group4 (≥3)	Regional lymph node (yes vs. no)	1
	PNI (PNI<49 vs. PNI≥49)	1
IPI+PNI		
Low (0-1)	Age (>60 years vs. ≤60 years)	1
Intermediate low (2)	Ann Arbor stage (III-IV vs. I-II)	1
Intermediate high (3)	ECOG score (≥2 vs. 0-1)	1
High (≥4)	Elevated LDH (yes vs. no)	1
	Distant extranodal involvement (≥2 vs. 0-1)	1
	PNI (PNI<49 vs. PNI≥49)	1
PINK+PNI		
Low risk (0)	Age (>60 years vs. ≤60 years)	1
Intermediate low (1)	Ann Arbor stage (III-IV vs. I-II)	1
Intermediate high (2)	Distant extranodal involvement (yes vs. no)	1
High risk (≥3)	Nonnasal disease (yes vs. no)	1
	PNI (PNI<49 vs. PNI≥49)	1

PNI, Prognostic nutritional index; ***LDH***, lactate dehydrogenase; ***ECOG***, Eastern Cooperative Oncology Group; ***IPI***, International Prognostic Index; ***KPI***, Korean Prognostic Index; ***PINK***, Prognostic index of natural killer lymphoma.

Supplementary Figures

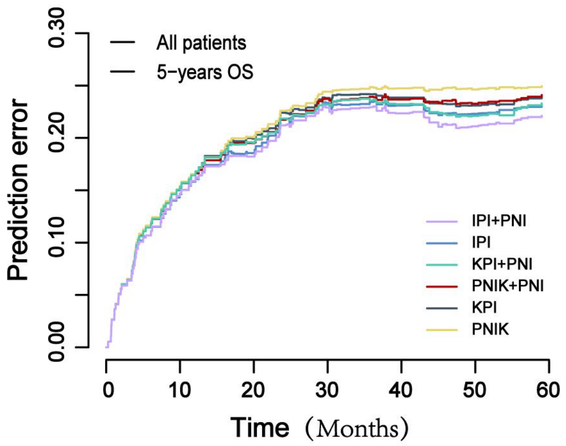

Supplementary Figure 1. Prognostic power evaluation of PNI. The IBS values of different models.
